# Outdoor Autonomous Navigation of a Tailless Flapping-Wing Micro Aerial Vehicle Using Low-Cost MEMS Sensors and GNSS Module

**DOI:** 10.3390/biomimetics11100725

**Published:** 2026-10-11

**Authors:** Jiahui Yuan, Yiming Xiao, Chaofeng Wu, Hanlin Yuan, Xiaosheng Wu, Wu Liu, Feng Cui

**Affiliations:** 1 National Key Laboratory of Advanced Micro and Nano Manufacture Technology, Shanghai Jiao Tong University, Shanghai 200240, China; yuanjiahui@sjtu.edu.cn (J.Y.); xiaoyiming0379@sjtu.edu.cn (Y.X.); 897124663@sjtu.edu.cn (C.W.); lin0207@sjtu.edu.cn (H.Y.); xswu@sjtu.edu.cn (X.W.); liuwu@sjtu.edu.cn (W.L.); 2 School of Integrated Circuits (School of Information Science and Electronic Engineering), Shanghai Jiao Tong University, Shanghai 200240, China

**Keywords:** tailless flapping-wing micro aerial vehicle, outdoor autonomous flight, MEMS/GNSS, error-state Kalman filter

## Abstract

Bio-inspired tailless flapping-wing micro aerial vehicles (FWMAVs) offer advantages of hovering flight, small size, and high maneuverability, showing potential for confined space missions. Outdoor autonomous flight remains challenging for tailless FWMAVs because of their strict payload and onboard-computing constraints. These constraints limit the use of high-precision sensors and computationally intensive perception methods, making reliable onboard state estimation difficult in outdoor environments. To address this issue, this paper presents an onboard autonomous flight scheme for a 31 g insect-inspired tailless FWMAV with a wingspan of 33 cm. Low-cost MEMS sensors and a GNSS module are used to obtain onboard motion and position information. An error-state Kalman filter (ESKF) fuses this measurement information to provide continuous estimates of the vehicle state. Based on the estimated state, a cascaded PID controller regulates the vehicle position and motion in a closed loop. All sensing, state estimation, and control functions are performed onboard without relying on external localization infrastructure. Outdoor experiments showed that the fused position estimates remained consistent with independently recorded real-time kinematic (RTK) GNSS measurements. The vehicle achieved autonomous position holding and stable flight along predefined paths under open-sky GNSS conditions. During three independent 20 m straight-line trajectory tracking flight trials, the average maximum lateral and vertical errors were 0.28 m and 0.10 m, respectively. A V-shaped altitude-varying trajectory tracking flight further demonstrated simultaneous horizontal and vertical motion, with a three-dimensional estimated-state-to-reference root mean square error (RMSE) of 0.0601 m. These results demonstrate the feasibility of fully onboard outdoor autonomous flight for tailless FWMAVs with sub-0.5 m wingspan under open-sky GNSS conditions.

## 1. Introduction

Micro aerial vehicles (MAVs) are generally classified into rotary-wing, fixed-wing, and flapping-wing types. Among them, rotary-wing MAVs, especially quadrotors, are widely used for autonomous flight due to their simple structures, high maneuverability, mature control methods, and relatively large payload capacity [1]. These advantages enable rotary-wing MAVs to integrate various onboard sensors and computing systems for navigation, perception, and various task operations. However, the high-speed rotating propellers of rotary-wing MAVs generate continuous aerodynamic noise, which increases the difficulty of achieving low-noise and highly concealed flight. In addition, the exposed rotating propellers may introduce safety concerns in certain application scenarios. In contrast, inspired by the flight mechanisms of insects and birds, flapping-wing micro aerial vehicles (FWMAVs) generate lift, thrust, and attitude control moments through periodic wing flapping motions. Their biomimetic appearance, small size, low noise, and high maneuverability provide unique advantages for lightweight aerial platforms in applications such as reconnaissance, environmental monitoring, and disaster search [2,3,4]. Therefore, the development of FWMAVs is of great research significance.

Bio-inspired flapping-wing vehicles are mainly divided into insect-inspired tailless flapping-wing vehicles and bird-inspired tailed flapping-wing vehicles. Bird-inspired tailed flapping-wing vehicles usually have larger wingspans and payload capacity. Their flight control is generally achieved by using the flapping mechanism for lift and propulsion, while tail wings or tail control surfaces assist attitude regulation. Therefore, they are more suitable for long-endurance cruising, wind-resistant flight, path tracking, and payload-carrying missions in outdoor environments. For example, HIT-Hawk and HIT-Phoenix [5,6,7], two kinds of robotic birds with wingspans beyond 2 m, integrate onboard flight controllers, MEMS sensors (inertial measurement unit (IMU), magnetometer and barometer, etc.), GNSS (Global Navigation Satellite System), and communication modules. They achieved outdoor autonomous or semi-autonomous flight, including takeoff, climbing, maneuvering, descent, and landing. The Dove [8,9], with a wingspan of 50 cm, used a similar approach and achieved fully autonomous flight and long-distance video transmission. However, their larger size, weight, and specific aerodynamic properties hinder their agility and maneuverability, making them less suited for operations in confined environments or for hovering flight. Compared with bird-inspired flapping-wing vehicles, insect- inspired tailless flapping-wing vehicles with hovering capability have great potential for miniaturization and agile flight. The tailless FWMAVs have smaller size, lower weight, and higher maneuverability, making them suitable for confined-space applications [10].

Inspired by the remarkable maneuverability of flying insects, tailless FWMAVs aim to reproduce the agile flight capabilities of these insects. However, due to the absence of conventional tail stabilizers, their attitude regulation mainly depends on differential flapping frequency and wing-surface mechanism adjustment. In addition, their payload, power, and computing resources are more limited, especially for tailless FWMAVs with wingspans less than 0.5 m. High-frequency vibration induced by wing flapping can also significantly degrade the measurement quality of onboard sensors. Therefore, translating the high flight maneuverability of tailless FWMAVs into practical autonomous flight systems remains difficult. At present, many untethered tailless FWMAVs still mainly rely on manual remote control. Because FWMAVs have lightweight, small inertia, and high sensitivity to wind disturbances, manual control requires considerable operator experience. This makes it difficult to meet the requirements of long-duration, repeatable, and complex missions [11]. Therefore, enabling FWMAVs to achieve autonomous perception, localization, and trajectory tracking is a key step toward practical applications. However, autonomous flight of tailless FWMAVs remains challenging due to their limited onboard payload capacity, computing resources, and energy supply. In addition, their nonlinear aerodynamic characteristics increase the difficulty of accurate state estimation and robust flight control [12].

Research on tailless flapping-wing FWMAVs has made some progress. However, very few of them are capable of autonomous outdoor flight. Currently, their autonomous flight relies mainly on onboard vision or external positioning systems. The DelFly series developed by Delft University of Technology integrates a miniature IMU, a barometer, and a customized vision module to achieve stable indoor flight and obstacle avoidance [13,14,15,16]. The tailless biomimetic flapping-wing vehicle, developed by Flapper Drones—a spin-off from Delft University of Technology—adopts an ultra-wideband (UWB) indoor localization system. An extended Kalman filter is used to fuse UWB and IMU measurements for indoor pose estimation. With additional drag compensation and ground effect mitigation, the system achieves a positioning accuracy of approximately 10 cm [17]. The BionicBee developed by Festo [18,19] uses an infrared camera network deployed around the flight space to form a high-precision indoor motion capture system. This system is combined with onboard IMUs to achieve multi-vehicle localization and coordination. These studies demonstrate the feasibility of autonomous flight for flapping-wing vehicles. However, their limitations are also clear. Vision-based methods usually require considerable onboard computing power, stable illumination, and image-processing modules. These requirements are difficult to satisfy on highly miniaturized platforms with limited payload capacity, computing resources, and energy supply [20]. External motion capture or UWB systems can provide high-precision pose information in controlled indoor environments, but they rely on pre-installed infrastructure and are difficult to extend directly to open outdoor environments. Fully onboard outdoor autonomous flight capability has not been demonstrated by existing indoor-oriented miniature FWMAVs. Furthermore, the limited onboard hardware resources of miniature FWMAVs preclude the direct adoption of autonomous flight solutions developed for conventional UAV platforms. This necessitates the development of miniaturized navigation and control strategies tailored to such resource-constrained platforms.

A feasible solution is to use onboard low-cost micro-electromechanical systems (MEMS) sensors together with a GNSS module for integrated navigation [21]. The IMU can provide angular velocity and acceleration measurements at a high frequency. It is suitable for capturing the rapidly changing attitude and short-term motion state of FWMAVs. The GNSS can provide long-term stable absolute position and velocity constraints, which are useful for suppressing the accumulation of inertial integration errors. However, low-cost IMU sensors suffer from bias drift and random noise. The GNSS has a relatively low output frequency and is susceptible to multipath effects, signal blockage, and electromagnetic interference [22]. Therefore, reliable state estimation requires effective fusion of complementary sensor information with consideration of sensor uncertainty.

Common MEMS/GNSS data fusion methods include complementary filtering, Kalman filtering, and their extended forms. Complementary filtering has a simple structure [23]. It can exploit the complementary characteristics between the high-frequency response of the IMU and the low-frequency absolute constraints of GNSS. However, its parameter selection usually depends on empirical tuning, and it is difficult to systematically describe sensor noise and state uncertainty. Under the combined effects of low-cost sensor noise, bias drift, and dynamic flight conditions, complementary filtering with fixed parameters may not guarantee long-term stability in position and attitude estimation. The extended Kalman filter (EKF) [24,25] performs recursive estimation of nonlinear system states, such as position, velocity, and attitude, by applying first-order linearization. It is widely used in integrated navigation. However, when the system has strong nonlinearity, large attitude errors, or unstable covariance propagation, direct linearization of the full state may introduce considerable linearization errors. This can further affect estimation accuracy and stability.

The error-state Kalman filter (ESKF) [26,27,28] decomposes the system state into a nominal state and an error state. Only the small error state is linearized. After each measurement update, the estimated error is injected into the nominal state, and the error state is reset. This formulation allows the filter to operate around a small-error working point throughout the estimation process, which improves numerical stability. It is especially suitable for attitude, velocity, and position fusion estimation using low-cost MEMS IMUs. Based on these advantages, this study integrates an ESKF into the onboard flight-control system of our self-developed tailless FWMAV [29]. The aim is to realize reliable onboard state estimation under low-rate GNSS updates and MEMS IMU drift. Outdoor experiments, including ground-based validation experiments and autonomous flight experiments, are conducted to validate the proposed approach.

The main contributions of this work are summarized as follows:

(1) An onboard autonomous flight system, integrating a 1.9 g flight-control board (with MEMS sensors integrated) and a 2.4 g GNSS module, is developed for a lightweight tailless FWMAV with a wingspan of 33 cm and a total mass of 31 g. Compared with existing tailless FWMAVs such as BionicBee, Flapper and DelFly, the proposed system demonstrates the feasibility of fully onboard outdoor autonomous flight using only low-cost MEMS sensors and a GNSS module under strict payload and computational constraints.
(2) A low-cost MEMS/GNSS fusion navigation framework is developed for miniature tailless FWMAVs. The proposed ESKF-based method enables real-time onboard estimation of position, velocity, and attitude using only lightweight MEMS sensors and GNSS fusion measurements, providing a practical solution for autonomous navigation of FWMAVs.
(3) A practical state-feedback cascaded PID control strategy for the fusion navigation framework is developed for outdoor flight control without requiring an accurate aerodynamic model. The proposed lightweight control strategy enables autonomous position holding, straight-line trajectory tracking and V-shaped altitude-varying trajectory tracking with low computational complexity. This demonstrates the feasibility of model-independent control for bio-inspired tailless FWMAVs with sub-0.5 m wingspan.

The remainder of this paper is organized as follows. Section 2 introduces the tailless FWMAV and its attitude control principle, onboard navigation and control system architecture, navigation fusion method, and trajectory tracking control method. Section 3 presents the experimental results and their analysis. Section 4 discusses the achievements and limitations of the experimental results, as well as the future work to be carried out. Section 5 concludes the paper.

## 2. System Architecture and Methods

This section presents a lightweight onboard autonomous navigation and trajectory-tracking scheme tailored for an insect-inspired tailless FWMAV. To achieve outdoor autonomous flight independent of external motion capture systems, a hierarchical navigation and control framework is constructed using low-cost MEMS sensors and a GNSS module. First, the mechanical structure of the vehicle and its integrated electronic system are introduced to establish the hardware foundation. Second, an Error-State Kalman Filter (ESKF) algorithm is developed for multi-sensor fusion, leveraging high-frequency IMU state propagation and low-frequency absolute constraints from a barometer and a GNSS module to yield continuous three-dimensional position and velocity states. Finally, a cascaded PID controller is designed to map position and path-tracking errors into baseline attitude commands, thereby achieving stable closed-loop trajectory control. The architectural layers and algorithmic implementations are detailed in the following subsections.

### 2.1. Tailless FWMAV and System Architecture

The flight experimental system used in this study consists of a self-developed tailless FWMAV [29], a remote controller, and a PC-based ground station. The remote controller sends throttle, attitude angle, and mode-switching commands to the aircraft through a 2.4 GHz wireless link. The PC-based ground station is used to receive real-time flight-control data, display the flight status, and send parameter configuration commands.

**Figure 1:**
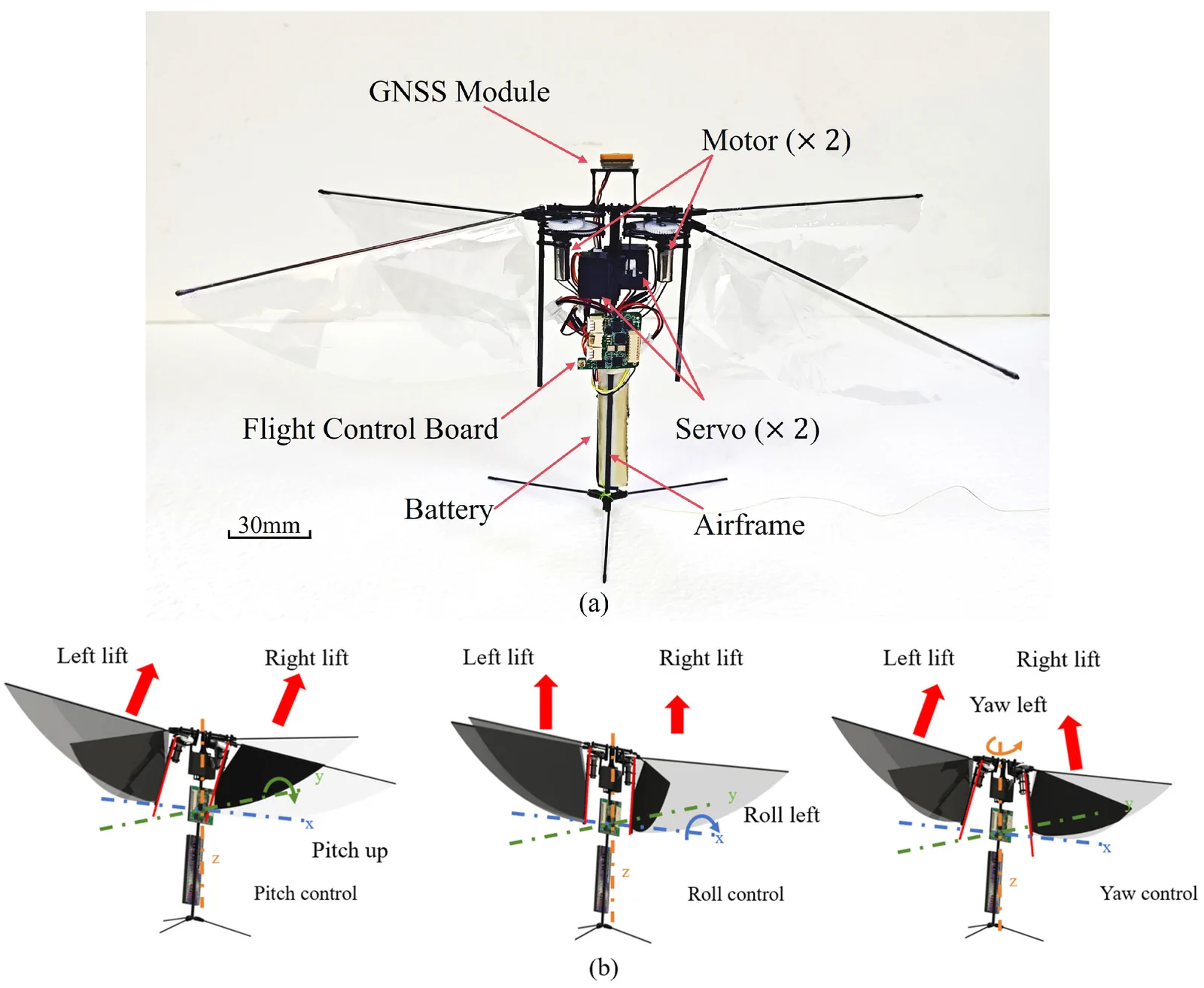
(**a**) Prototype of the tailless FWMAV equipped with a GNSS module. (**b**) Schematic diagrams of the three-axis attitude control principle, where the blue, green, and orange dotted lines indicate the *x*, *y*, and *z* axes, respectively.

The tailless FWMAV prototype [29] is shown in Figure 1a. It consists of two flapping mechanisms, two servos, a flight control board, a GNSS module, a lithium battery, and an airframe. It has a wingspan of 33 cm and a total mass of 31 g. The FWMAV adopts a four-wing configuration, with one flapping mechanism on each side. Each flapping mechanism contains a pair of wings. The two flapping mechanisms are driven by independent coreless motors. The motor rotation is converted into reciprocating wing flapping through a reduction gearbox and a linkage mechanism.

To achieve attitude control, the FWMAV relies on the lift variation generated by the two flapping mechanisms and the directional adjustment of their stroke planes to produce control torques [29]. As shown in Figure 1b, the aircraft generates roll control torque by adjusting the speed difference between the left and right motors. The two servos adjust the linkage mechanism and wing attitude, thereby producing pitch- and yaw-related control torques. This stroke-plane lift-vector control method enables the aircraft to achieve three-axis attitude regulation without a tail. More details on the structural design and attitude control method of this tailless FWMAV can be found in [29].

**Figure 2:**
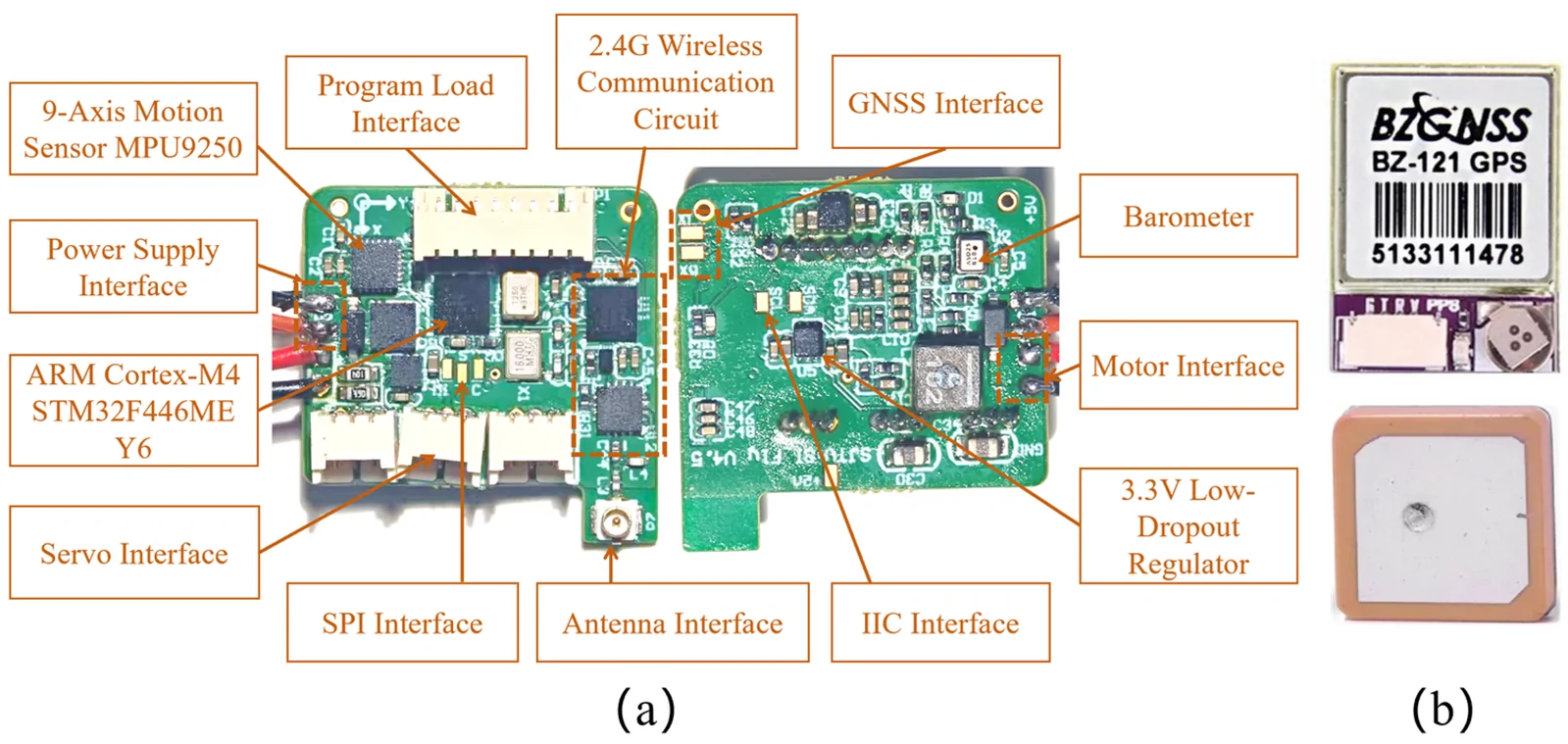
(**a**) Flight control circuit board module, shown from both sides. (**b**) GNSS module, shown from both sides.

This study uses a self-developed miniaturized flight control circuit board module, as shown in Figure 2a. The printed circuit board (PCB) has dimensions of 23.8 mm × 23.8 mm × 0.4 mm. The whole control module weighs only 1.9 g. It uses an STM32F446MEY6 as the main control chip and integrates an MPU9250 motion sensor, an ISP06 barometer, wireless communication circuitry, two low-dropout (LDO) regulators, a DC-DC boost converter, two motor drivers, and several interfaces. The MPU9250 sensor is used to obtain motion data from the IMU (including a three-axis gyroscope and a three-axis accelerometer) and a three-axis magnetometer. These measurements provide high-frequency motion information for attitude estimation and inertial propagation. It should be noted that due to the electromagnetic interference generated by the flapping mechanism, the data from the three-axis magnetometer in the MPU9250 is not used in this study. The ISP06 barometer is used for altitude measurement. The 2.4 GHz wireless communication circuitry mainly uses an nRF51822-CDAB chip for data transmission between the aircraft and the remote controller.

The GNSS module, model BZ121, weighs only 2.4 g and has dimensions of 16 mm × 12 mm × 4.6 mm, as shown in Figure 2b. It is mounted on top of the airframe and connected to the flight control board through the GNSS interface. The module uses a UBX-M10050 GNSS chip and provides three-dimensional position, velocity, and altitude data in outdoor environments.

This hardware combination does not rely on an indoor motion capture system or a high-computing-power vision module. It satisfies the requirements of flapping-wing micro platforms for low-mass, low-power, and low-cost onboard sensing.

**Figure 3:**
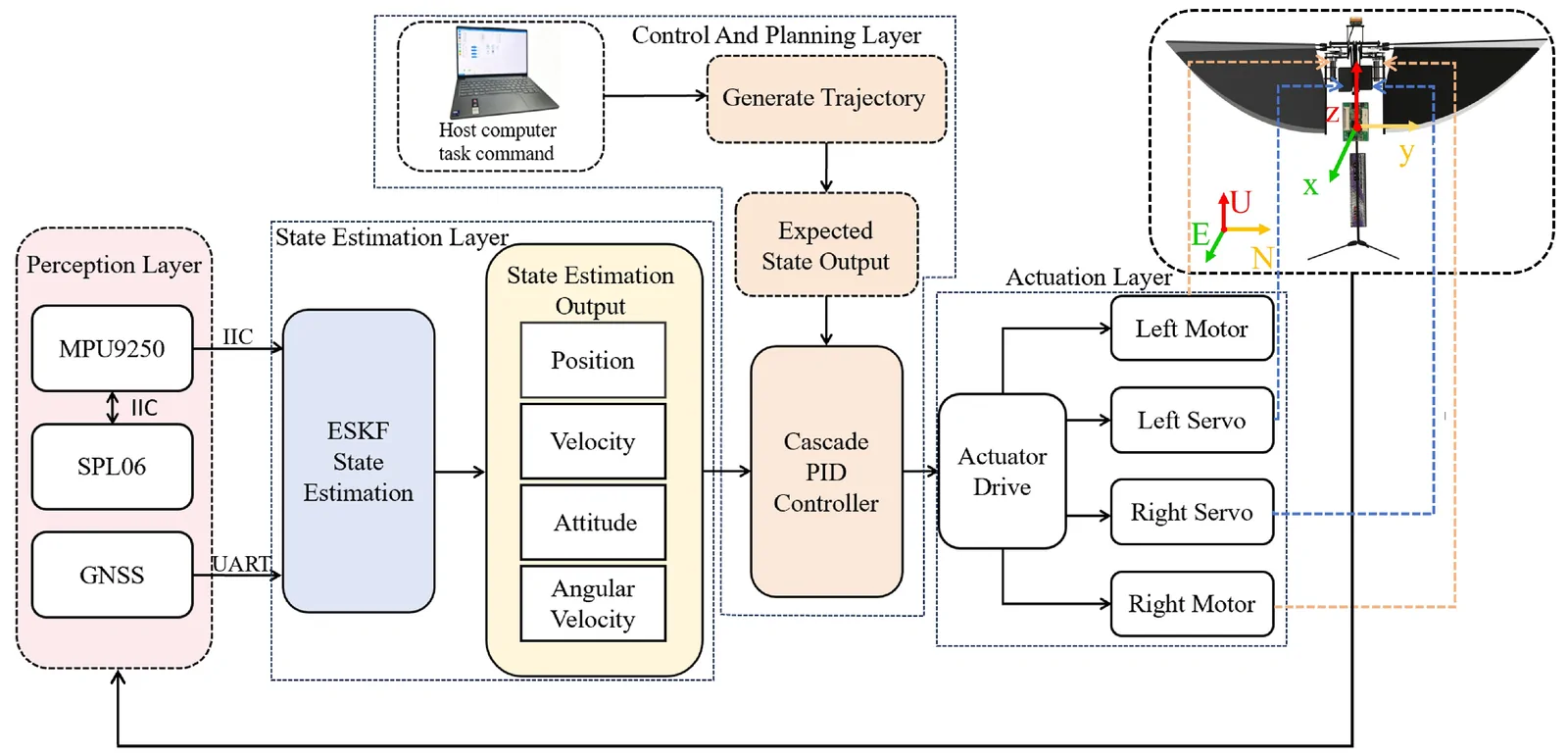
Overall architecture of the onboard navigation and control system. ESKF: Error-State Kalman Filter; PID: Proportional–Integral–Derivative controller; ENU: East–North–Up coordinate system. The black arrows indicate signal flow, while the colored dashed lines represent the connections between the control outputs and the corresponding actuators of the FWMAV.

Figure 3 shows the overall architecture of the onboard navigation and control system. The system mainly consists of four layers: the perception layer, the state estimation layer, the control and planning layer, and the actuation layer. First, the IMU, GNSS module, and barometer in the sensing layer collect raw sensor data. Then, the state estimation layer performs multi-sensor fusion using the ESKF and estimates the position, velocity, and attitude of the aircraft. The task generation module in the ground station sends autonomous flight task commands. In the control and planning layer, the cascaded PID controller generates desired state values according to hovering or trajectory tracking tasks. It then calculates the errors between the desired states and the estimated states, and generates control commands. Finally, the actuation layer adjusts the motor speeds of the flapping mechanisms or the servo output angles according to the control commands. In this way, the flight attitude is changed, and position holding and trajectory tracking are achieved.

This study uses a local East–North–Up (ENU) coordinate system (also known as an inertial coordinate system) to describe the translational motion of the aircraft. The *X*-axis, namely the *E*-axis, points east. The *Y*-axis, namely the *N*-axis, points north. The *Z*-axis, namely the *U*-axis, points upward. The body coordinate system is fixed to the aircraft body and is used to describe angular velocity, acceleration, and attitude motion. The longitude and latitude data output by the GNSS module are converted into local ENU coordinates before being used in state estimation and control. This ensures consistency with the IMU propagation results and the reference trajectory.

### 2.2. ESKF-Based MEMS/GNSS State Estimation

To obtain continuous position, velocity, and attitude estimates of the FWMAV under low-cost MEMS sensor conditions, this study uses the ESKF to fuse IMU, GNSS, and barometer measurements. Unlike the conventional EKF, which directly linearizes the full state, the ESKF separates the system state into a nominal state and an error state. The ESKF only linearizes the small error state, which improves the numerical stability of the estimation process.

The nominal state is defined as

(1) $$x={\left[p\;v\;q\;b_{g}\;b_{a}\right]}^{T}$$

where ***p***, ***v***, q, $b_{g}$ and $b_{a}$ denote the position, velocity, attitude quaternion, gyroscope bias, and accelerometer bias, respectively. The nominal state represents the estimated motion state of the aircraft.

The corresponding 15-dimensional error state is defined as

(2) $$\delta x={\left[\delta p^{n}\;\delta v^{n}\;\delta q\;\delta b_{g}\;{\delta b}_{a}\right]}^{T}$$

where δ***p*^n^** and δ***v*^n^** denote the position error and the velocity error in the inertial frame. δ***q*** denotes the small-angle attitude error. ${\delta b}_{g}\;\mathrm{and}\;\delta b_{a}$ are the gyroscope bias and accelerometer errors, respectively. The error state represents small deviations caused by inertial integration errors and sensor bias uncertainties.

Based on the above definitions, the description of the ESKF-based MEMS/GNSS state estimation process can be divided into four parts. First, the IMU kinematics and error model are introduced. Then, the GNSS and barometer measurement models are described. Next, the error-state Kalman filter is presented. Finally, the error-state estimate is injected into the nominal state, and the error state is reset.

#### 2.2.1. IMU Kinematics and Error Model

The IMU mainly consists of a three-axis accelerometer and a three-axis gyroscope. Its measurement model can be expressed as

(3) $$a_{m}=a+b_{a}+n_{a}$$

(4) $$\omega_{m}=\omega +b_{g}+n_{g}$$

where $a_{m}$ and $\omega_{m}$ are the measured acceleration and angular velocity, respectively. a and ω denote the true specific force and true angular velocity, respectively. $b_{a}$ and $b_{g}$ are the accelerometer bias and gyroscope bias, respectively. $n_{a}$ and $n_{g}$ denote zero-mean Gaussian white noises of the accelerometer and gyroscope, respectively.

The sensor noise characteristics were considered in the ESKF covariance configuration. The main noise parameters of the onboard sensors, including IMU noise densities, bias instability, and barometer noise, are obtained from the corresponding sensor datasheets [30,31] and the literature [32], and are summarized in Table 1. The process noise covariance matrix ***Q*** and measurement noise covariance matrix ***R*** were configured according to the sensor characteristics and validated through flight tests.

Due to the periodic flapping motion, high-frequency fluctuations may appear in the accelerometer measurements. Therefore, the measured acceleration is preprocessed before being used for inertial propagation in the ESKF. A dynamic moving average filter is applied to reduce the influence of high-frequency measurement noise.

(5) $$a_{f}[n]=\frac{1}{N}\sum_{k=0}^{N-1}a_{m}[n-k]$$

where $a_{f}[n]$ denotes the filtered acceleration used for inertial propagation, and N is the moving window length. In this study, N=53. This preprocessing is performed before the ESKF propagation step and is independent of the state estimation framework.

Strapdown inertial navigation calculation includes attitude, velocity, and position updates. The attitude is represented by the quaternion ***q***. The rotation matrix from the body frame to the inertial frame is denoted as ***R***(***q***). After gyroscope bias compensation, the bias-compensated angular velocity is written as

(6) $$\hat{\omega }=\omega_{m}-{\hat{b}}_{g}$$

The attitude quaternion is updated as

(7) $${\hat{q}}_{k+1}={\hat{q}}_{k}⨂\delta q(\hat{\omega }∆t)$$

where ⨂ denotes quaternion multiplication, $\delta q(\hat{\omega }∆t)$ is the incremental quaternion constructed from the angular increment, Δ*t* is the IMU sampling period, and *k* is the discrete sampling index.

After filtering and bias compensation, the specific force can be expressed as

(8) $$\hat{a}=a_{f}-{\hat{b}}_{a}$$

After it is transformed into the inertial frame, the velocity and position are updated as

(9) $${\hat{v}}_{k+1}={\hat{v}}_{k}+[R\left({\hat{q}}_{k}\right)\hat{a}+g]∆t$$

(10) $${\hat{p}}_{k+1}={\hat{p}}_{k}+{\hat{v}}_{k}∆t+\frac{1}{2}[R\left({\hat{q}}_{k}\right)\hat{a}+g]∆t^{2}$$

where *g* is the gravitational acceleration in the inertial frame.

#### 2.2.2. GNSS and Barometer Measurement Models

GNSS provides position and velocity measurements in the inertial frame. The GNSS measurement model can be expressed as

(11) $$p_{GNSS}=p+n_{p}$$

(12) $$v_{GNSS}=v+n_{v}$$

where $p_{GNSS}$ and $v_{GNSS}$ are the position and velocity measured by GNSS, respectively. p and v denote the true position and velocity of the aircraft. $n_{p}$ and $n_{v}$ are the GNSS position and velocity measurement noises, respectively.

The barometer provides altitude information. The barometer measurement model is:

(13) $$z_{b}=h\;+\;n_{b}$$

where h is the vertical position component and $n_{b}$ represents barometer noise.

For GNSS position and velocity measurements, the observation matrix can be written as

(14) $$H=\left[\begin{array}{ccccc} I_{3} & 0 & 0 & 0 & 0\\ 0 & I_{3} & 0 & 0 & 0 \end{array}\right]$$

For barometer altitude measurements, only the vertical position error is observed. Therefore, the observation matrix is defined as

(15) $$H_{b}=\left[\begin{array}{ccccc} 0 & 0 & 1 & 0 & 0 \end{array}\right]$$

where the nonzero element corresponds to the vertical position component.

Since GNSS usually outputs longitude, latitude, and altitude, the aircraft position is converted into a local East–North–Up (ENU) frame referenced to ($L_{0},B_{0},h_{0}$). The ENU coordinates of the aircraft position are approximated by

(16) $$x=\left(L-L_{0}\right)cosB_{0}R_{E}$$

(17) $$y=\left(B-B_{0}\right)\cdot R_{E}$$

(18) $$z=h-h_{0}$$

where $R_{E}$ is the Earth’s radius; x,y and z denote the local east, north, and up position components, respectively; *L*, *B*, and *h* denote longitude, latitude, and altitude, respectively. This local coordinate system is used for the subsequent ESKF update and position control.

No explicit outlier rejection method is applied to GNSS measurements in this study. The uncertainty of GNSS position and velocity observations is represented by the measurement noise covariance matrix R, which determines the contribution of GNSS measurements during the ESKF update step.

#### 2.2.3. Error-State Kalman Filter

The main idea of the ESKF is to filter the error state instead of directly filtering the full state. The continuous-time error-state equation can be written as

(19) $$\delta \dot{x}=F\delta x+Gn$$

where ***F*** is the error-state transition matrix, ***G*** is the noise driving matrix, and ***n*** is the system process noise.

For the defined error state, the transition matrix is:

(20) $$F=\left[\begin{array}{ccccc} 0 & I_{3} & 0 & 0 & 0\\ 0 & 0 & {\left[f\right]}_{\times } & 0 & C_{b}^{n}\\ 0 & 0 & 0 & -C_{b}^{n} & 0\\ 0 & 0 & 0 & 0 & 0\\ 0 & 0 & 0 & 0 & 0 \end{array}\right]$$

where $f={\left[f_{e},f_{n},f_{u}\right]}^{T}$ represents the specific force in the inertial frame; the skew-symmetric matrix ${\left[f\right]}_{\times }$ is defined as

(21) $${\left[f\right]}_{\times }=\left[\begin{array}{ccc} 0 & -f_{u} & f_{n}\\ f_{u} & 0 & -f_{e}\\ -f_{n} & f_{e} & 0 \end{array}\right]$$

which corresponds to the implementation in the onboard estimator.

For onboard implementation, the continuous-time error-state model is discretized using the first-order Euler method. The discrete-time state transition matrix is obtained as

(22) $$\phi_{k}=I+F_{k}∆t$$

The error covariance prediction is given by

(23) $$P_{k|k-1}=\phi_{k}P_{k-1}{\phi_{k}}^{T}+Q_{k}$$

where $Q_{k}$ is the discrete process-noise covariance matrix. When GNSS or barometer measurements are available, the measurement residual is calculated as

(24) $$r_{k}=z_{k}-h({\hat{x}}_{k|k-1})$$

where $z_{k}$ represents the actual GNSS or barometer measurement, and $h({\hat{x}}_{k|k-1})$ is the predicted measurement calculated from the predicted nominal state.

The Kalman gain is calculated as

(25) $$K_{k}=P_{k|k-1}H^{T}{\left(HP_{k|k-1}H^{T}+R\right)}^{-1}$$

The estimated error state is obtained as

(26) $$\delta {\hat{x}}_{k}=K_{k}r_{k}$$

The covariance is updated as

(27) $$P_{k}=\left(I-K_{k}H\right)P_{k|k-1}{\left(I-K_{k}H\right)}^{T}+K_{k}R{K_{k}}^{T}$$

This form improves the numerical stability of the covariance update.

#### 2.2.4. Error Injection and Reset

After the error-state estimate value is obtained, it is injected into the nominal state. The position, velocity, and biases are corrected by addition:

(28) $${\hat{p}}_{k}\leftarrow {\hat{p}}_{k}+\delta {\hat{p}}_{k}$$

(29) $${\hat{v}}_{k}\leftarrow {\hat{v}}_{k}+\delta {\hat{v}}_{k}$$

(30) $${\hat{b}}_{a,k}\leftarrow {\hat{b}}_{a,k}+\delta {\hat{b}}_{a,k}$$

(31) $${\hat{b}}_{g,k}\leftarrow {\hat{b}}_{g,k}+\delta {\hat{b}}_{g,k}$$

The attitude error is corrected using a small-angle quaternion:

(32) $$\delta q\approx {\left[1,\frac{1}{2}\delta {\hat{\theta }}^{T}\right]}^{T}$$

(33) $${\hat{q}}_{k}\leftarrow \delta q⊗{\hat{q}}_{k}$$

After error injection, the error state is reset to zero:

(34) $$\delta {\hat{x}}_{k}\leftarrow 0$$

Through the above nominal-state prediction, error covariance propagation, GNSS/barometer measurement update, error injection, and state reset, the ESKF continuously compensates for inertial integration errors and sensor bias drift in low-cost MEMS sensors.

### 2.3. Autonomous Navigation Flight Control Using Cascaded PID

To achieve autonomous flight of the tailless FWMAV, a state feedback-based cascaded PID control method is designed in this study. Although nonlinear or adaptive control methods may provide improved disturbance rejection and control performance under certain conditions, they usually require accurate aerodynamic modeling, reliable parameter identification, or additional computational resources. However, the flight dynamics of the tailless FWMAV involve nonlinear aerodynamic effects, and an accurate dynamic model is difficult to establish due to the complexity of flapping-wing motion. Moreover, the onboard resources of this lightweight platform are limited. Therefore, this study adopts a cascaded PID controller with a simple structure and easy engineering implementation for position holding and trajectory tracking.

**Figure 4:**
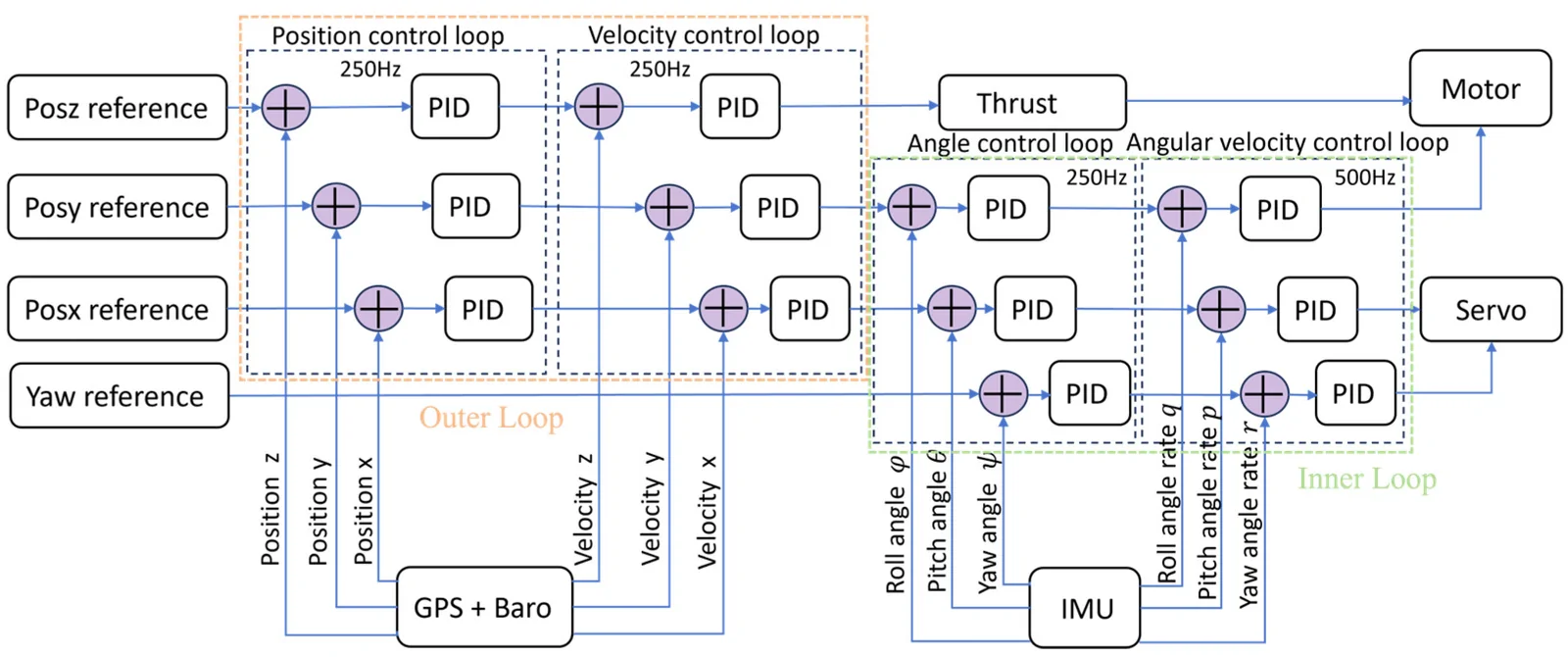
Overall block diagram of the aircraft position and attitude control system. The frequency indicated is the update frequency of the corresponding control loop. The purple circles containing a “+” symbol represent summing nodes used to calculate the control error for the two input parameters (associated with the two input arrows).

Considering the strong coupling between position and attitude, and their different response requirements, a cascaded control structure is adopted, as shown in Figure 4. The controller consists of four loops: position, velocity, attitude-angle, and angular-velocity loops. The position loop generates velocity commands according to the position error between the desired position and the estimated position from the ESKF. The velocity loop further converts these commands into attitude references. The attitude-angle loop tracks the desired attitude and generates angular velocity commands, while the angular-velocity loop produces actuator commands according to the angular-rate error.

In this cascaded control structure, the position and velocity loops act as outer loops for position regulation, while the angle and angular-velocity loops serve as inner loops for attitude stabilization. The outputs of each outer loop are used as reference inputs for the subsequent control loop, allowing position errors to be gradually converted into attitude adjustments and actuator commands.

In the controller design, *i* denotes the discrete sampling instant. For any control channel, the error at the *i*-th sampling instant is defined as

(35) $$e_{i}\;={\;r}_{i}-y_{out,i}$$

where $r_{i}$ is the reference input, $y_{out,i}$ is the feedback output of the system. The PID controller generates the control output according to the current error, accumulated error, and error variation rate. Its discrete form can be written as

(36) $$u_{i}=K_{p}e_{i}+K_{i}\sum_{j=0}^{i}e_{j}∆t+K_{d}\frac{e_{i}-e_{i-1}}{∆t}$$

where $u_{i}$ is the control output at the *i*-th sampling instant. $K_{p}$, $K_{i}$ and $K_{d}$ are the proportional, integral, and derivative gains, respectively. Δ*t* is the controller sampling period.

The PID gains were tuned experimentally through iterative flight tests following the cascaded control structure. The inner angular-rate loop was tuned first to obtain fast and stable responses to rate commands, followed by the attitude angle loop to achieve accurate attitude tracking. Subsequently, the velocity and position loop gains were adjusted sequentially to improve trajectory tracking performance. Integral gains were only introduced in channels with noticeable steady-state errors, while derivative gains were used to suppress overshoot and transient oscillations. The final PID parameters used in the experiments are summarized in Table 2.

The attitude and position controllers operate at different frequencies according to their dynamic response requirements. The angular-rate loop runs at 500 Hz, while the angle loop, velocity loop, and position loop all run at 250 Hz. The high-frequency angular rate loop is matched to the IMU update rate and uses IMU measurements for rapid attitude stabilization. The position-related loops use the continuously propagated state estimates from the ESKF. In the ESKF, IMU measurements support high-frequency (500 Hz) state propagation, while GNSS measurements at 10 Hz provide periodic position corrections. Therefore, the position controller can operate at 250 Hz using the propagated ESKF states between successive GNSS updates, while the lower-rate GNSS measurements provide long-term position constraints.

The mapping from controller outputs to actuator actions is illustrated in Figure 1b. At the actuation layer, the controller outputs are converted into motor and servo commands through a control allocation strategy. The thrust and roll control outputs are mapped to the flapping motors. The thrust command is distributed equally to the two motors, while the roll control output introduces an opposite offset to the left and right motor commands to generate differential speed for roll control. The pitch and yaw control outputs are converted into servo deflection commands. The pitch command produces differential servo deflections, while the yaw command produces coupled servo deflections to generate the corresponding control moments.

In this study, position holding and trajectory tracking are both described as position reference tracking problems in the navigation coordinate system. Let the desired position be $p_{d}(t)$. When $p_{d}(t)$ is constant, the control task corresponds to position holding. When $p_{d}(t)$ varies with time or is updated according to a waypoint sequence, the control task corresponds to trajectory tracking. Therefore, hovering, straight-line trajectory and V-shaped altitude-varying tracking tasks can be implemented within the same cascaded control framework, which consists of a position outer loop and an attitude inner loop.

## 3. Experimental Results and Analysis

This section presents the experimental validation of the proposed navigation and control system. First, a ground-based validation experiment [6] was conducted to evaluate the trajectory continuity and estimation performance of the developed ESKF algorithm, where RTK GNSS measurements were used as the reference. Second, the multi-sensor fusion algorithm was fully integrated into the 31 g tailless FWMAV for outdoor autonomous flight tests. Through autonomous position-holding, straight-line and V-shaped altitude-varying trajectory tracking missions, the closed-loop position-holding and trajectory tracking performance of the system was experimentally evaluated. The detailed results and analysis are presented below.

### 3.1. Ground-Based Validation of the MEMS/GNSS Fusion Navigation Algorithm

To verify the effectiveness of the proposed MEMS/GNSS fusion navigation algorithm in a real outdoor environment, a ground-based validation experiment was conducted at the sports field. The experiment was carried out at the South Campus Stadium of Shanghai Jiao Tong University. The site is open, and the GNSS signal is relatively stable. Therefore, it is suitable for preliminary validation of the low-cost MEMS/GNSS fusion navigation algorithm.

During the experiment, the operator held the aircraft and a high-precision RTK GNSS module (UM982) receiver and walked once around the running track of the sports field, finally returning to a position near the starting point. The running track is approximately a closed elliptical path. The ground station synchronously recorded the raw IMU measurements, the position and velocity information output by the GNSS module, and the three-axis position, velocity, and attitude estimates output by the onboard ESKF algorithm. The RTK GNSS module was used to independently record the reference position trajectory. Its RTK positioning accuracy is 0.8 cm + 1 ppm (RMS) horizontally and 1.5 cm + 1 ppm (RMS) vertically. This test was used to evaluate the continuity and stability of the MEMS/GNSS fusion estimation under outdoor motion conditions.

**Figure 5:**
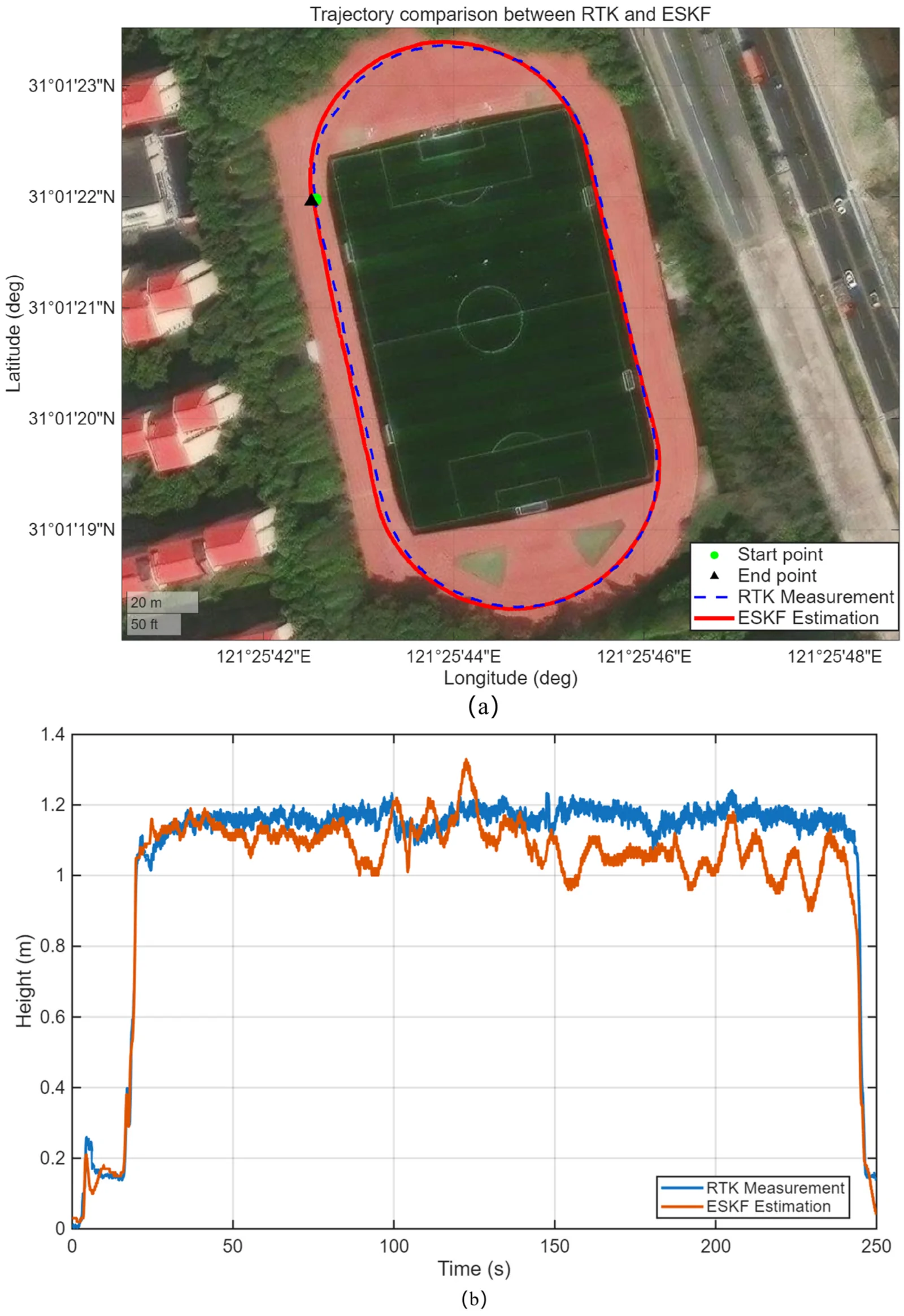
RTK-GNSS measured trajectory and ESKF estimated trajectory in the experiment where the operator held the aircraft and walked once around the sports field: (**a**) Comparison of 2D Trajectories obtained by RTK GNSS measurement and ESKF estimation; (**b**) Comparison of measured RTK GNSS altitude and ESKF estimated altitude.

Figure 5a shows the map overlay of the RTK-measured trajectory and the ESKF estimated trajectory during the ground-based validation experiment. The two trajectories are mainly distributed along the running track. The starting and ending positions are close to each other. The trajectory shape is also consistent with the predefined closed-loop walking path. This indicates that the experimental data can represent the predefined closed-loop motion around the field.

It can be seen that the ESKF estimated trajectory remains generally consistent with the RTK-measured trajectory throughout the continuous loop motion. No obvious jump or divergence occurs during the continuous loop motion. Since the GNSS output frequency is relatively low, while the IMU provides high-frequency short-term state propagation, the ESKF fusion result can maintain continuous position estimates between adjacent GNSS updates. This improves the temporal continuity and dynamic stability of the navigation result.

During the ground-based validation experiment, the operator carried both the RTK-GNSS receiver and the FWMAV by hand at approximately the same height while walking. Since the two devices were not rigidly mounted on the body, unavoidable hand motion and differences in holding posture introduced additional relative-height deviations during walking. Nevertheless, the altitude estimated by the ESKF remained generally consistent with the independently measured RTK-GNSS altitude and followed the main altitude variations throughout the experiment, as shown in Figure 5b.

The RMSE values between the ESKF estimates and RTK GNSS measurements in the east, north and altitude directions are 0.8780 m, 0.6368 m and 0.993 m, respectively. These RMSE values reflect the horizontal and vertical positioning deviations of the ESKF estimates with respect to the actual motion trajectory, indicating meter-level accuracy in both horizontal and vertical components.

In summary, the ground-based validation experiment verifies the effectiveness of the proposed ESKF-based fusion navigation algorithm under outdoor motion conditions. By combining high-frequency IMU propagation with low-frequency absolute constraints from GNSS and barometer measurements, the proposed fusion method provides continuous state feedback for subsequent outdoor autonomous flight control of the tailless FWMAV.

### 3.2. Outdoor Autonomous Flight Tests

After validating the MEMS/GNSS fusion navigation algorithm, the proposed navigation and control method was further integrated into the self-developed insect-like tailless FWMAV for outdoor autonomous flight tests. The tests were conducted in an outdoor environment with an ambient temperature of approximately 27 °C and a wind speed below 1.5 m/s. The flight experiments mainly included position-holding flight, straight-line trajectory tracking, and V-shaped altitude-varying trajectory tracking flight. These tests were used to evaluate the position-holding capability and horizontal path-tracking capability of the system, respectively.

The flight power of the tailless FWMAV mainly comes from periodic wing flapping. Its aerodynamic forces therefore have clear periodic and unsteady characteristics. In addition, due to the absence of conventional tail stabilizers, the attitude of the aircraft is mainly regulated by the flapping mechanisms and servos. The roll, pitch, and yaw dynamics are coupled due to the flapping-based attitude control mechanism. Therefore, outdoor flight tests can not only verify the usability of the state estimation algorithm, but also examine whether the low-cost MEMS/GNSS fusion navigation results can meet the closed-loop control requirements of the tailless FWMAV.

#### 3.2.1. Autonomous Position-Holding Flight

To verify the position-holding performance of the system, a flight experiment with a predefined position was conducted. The vehicle was commanded to reach and maintain the position setpoint. The control system calculated the deviation between the current position and the target point in real time based on the position and velocity estimates from the ESKF. The cascaded PID controller then adjusted the aircraft attitude and the outputs of the flapping actuators to keep the aircraft near the target point. This experiment was mainly used to evaluate the basic stability and anti-drift capability of the system when no time-varying path command was given.

**Figure 6:**
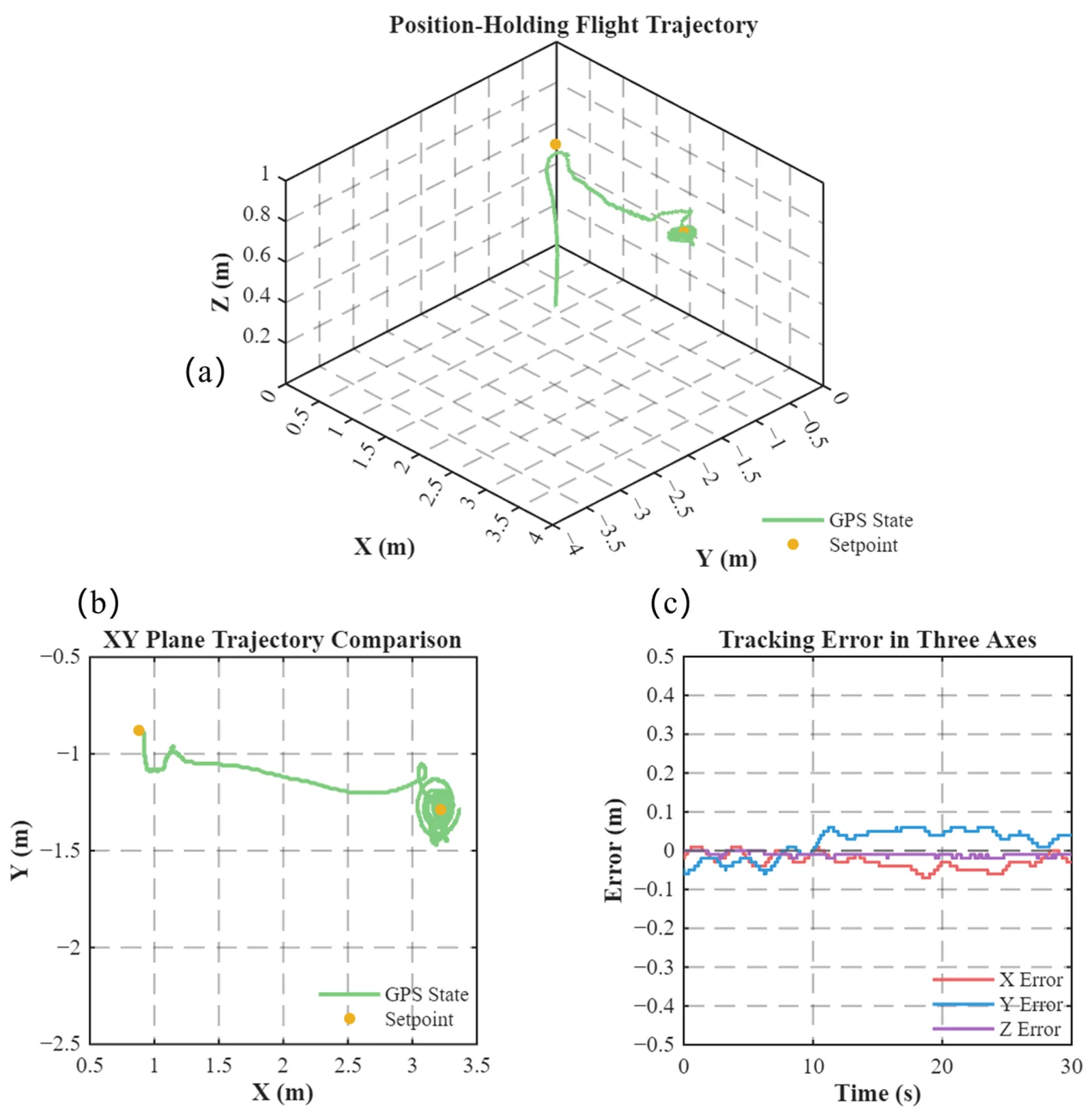
Position-holding hovering flight trajectory of the tailless FWMAV: (**a**) Three-dimensional trajectory based on the fusion estimation state; (**b**) XY plane trajectory; (**c**) Tracking error in three axes.

Figure 6a and Supplementary Video S1 show the trajectory of the position-holding experiment. The trajectory indicates that the aircraft first took off and approached the first setpoint (0.88 m, −0.88 m, 0.80 m). Then, according to the command from the ground station, it flew to the second setpoint (3.22 m, 1.29 m, 0.84 m) and hovered near this position. During the position-holding process, the aircraft moved around the target point and did not show continuous drift in a single direction.

The X-Y plane trajectory in Figure 6b further shows that the horizontal motion of the aircraft was mainly concentrated within a certain range around the target point. This indicates that the position loop continuously corrected the horizontal deviation based on the position estimates from the ESKF. Figure 6c shows the errors of the estimated X, Y, Z positions relative to the setpoint over the 30 s fixed-point flight duration. The error curves show certain fluctuations due to periodic flapping aerodynamics, short-term GNSS noise, and external wind disturbances. However, the overall errors remained bounded, and no obvious divergence occurred near the hovering target point.

**Figure 7:**
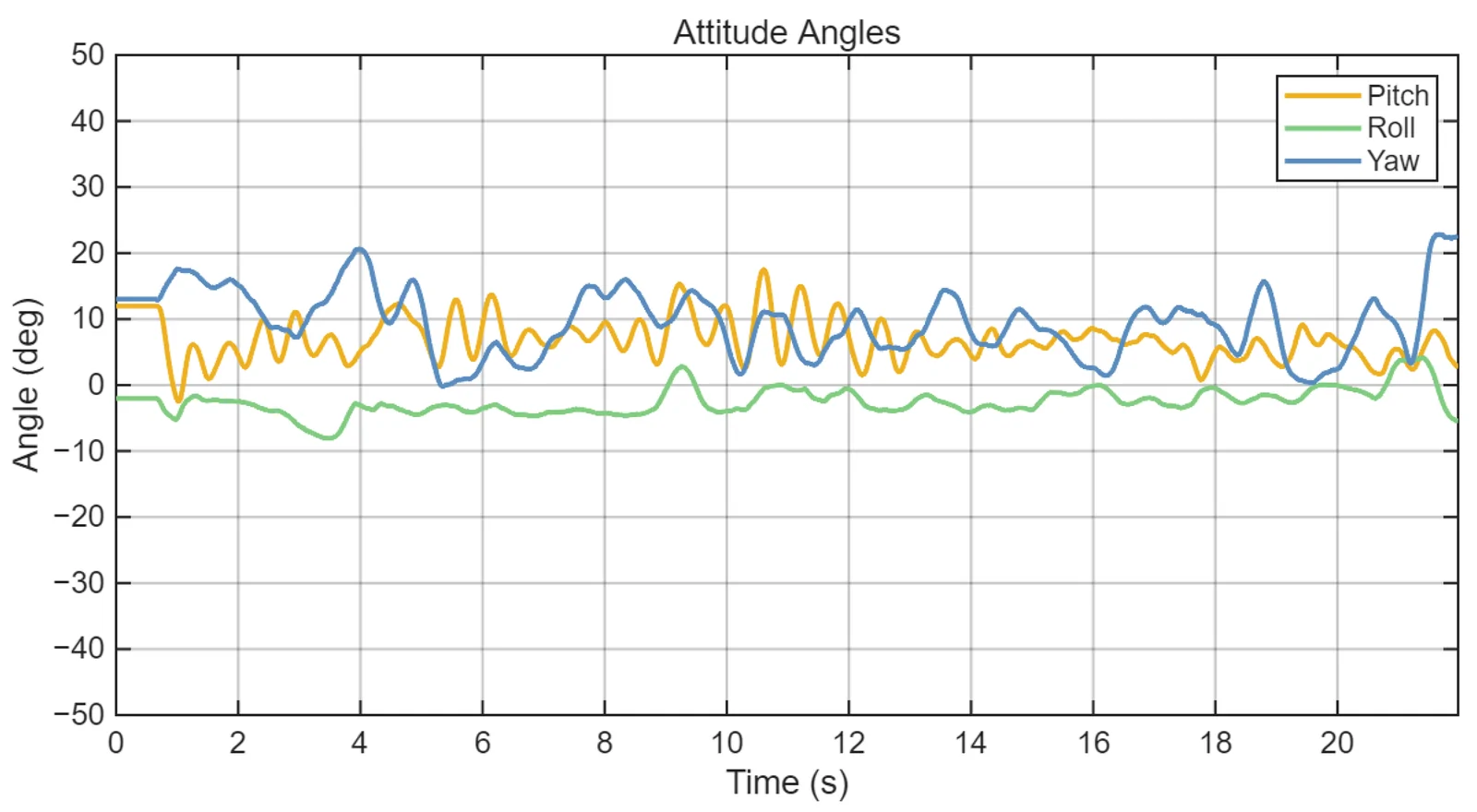
Attitude angle variations over a period of time during the Position-Holding flight.

A representative position-holding hovering flight segment was selected for attitude analysis, as shown in Figure 7. The attitude angle curves show that the aircraft maintained its attitude within a limited range during autonomous position holding. No continuous divergence or large oscillation was observed. This indicates that the attitude control loop could provide stable support for the position-holding task. The yaw angle showed some fluctuations, but it did not diverge rapidly. This suggests that the aircraft could still maintain basic heading stability under external disturbances.

The position-holding experiment shows that the proposed low-cost MEMS/GNSS fusion navigation and cascaded PID control method can support basic position holding for the tailless FWMAV. In outdoor environments, position holding is the basis for autonomous trajectory tracking. Stable position holding is a prerequisite for autonomous trajectory tracking and waypoint navigation.

#### 3.2.2. Straight-Line Trajectory Tracking Experiment

Based on the Position-holding experiment, a straight-line trajectory tracking experiment was further conducted to verify the autonomous path-following capability of the proposed navigation and control system. Compared with position holding, straight-line tracking requires the aircraft to maintain stable attitude and altitude while continuously moving along a predefined direction. It also requires the system to suppress the lateral deviation from the target path during flight. Therefore, this experiment can more directly reflect the application capability of the system in outdoor autonomous flight tasks.

In the experiment, a target straight-line path with a length of 20 m was predefined. The aircraft performed state feedback control based on the position and velocity estimates from the ESKF, so that it could fly along the target straight-line direction. The entire flight lasted 30 s. To evaluate the tracking performance, the lateral tracking error in the horizontal plane and the vertical tracking error relative to the desired altitude were calculated. The lateral tracking error is defined as the perpendicular distance from the current horizontal position of the aircraft to the projection of the target line. The vertical tracking error is defined as the difference between the actual altitude and the desired altitude. In this way, the horizontal path-following performance and altitude-hovering performance can be analyzed separately. To evaluate the repeatability of the trajectory tracking performance, three independent straight-line tracking trials were conducted under the same experimental conditions.

**Figure 8:**
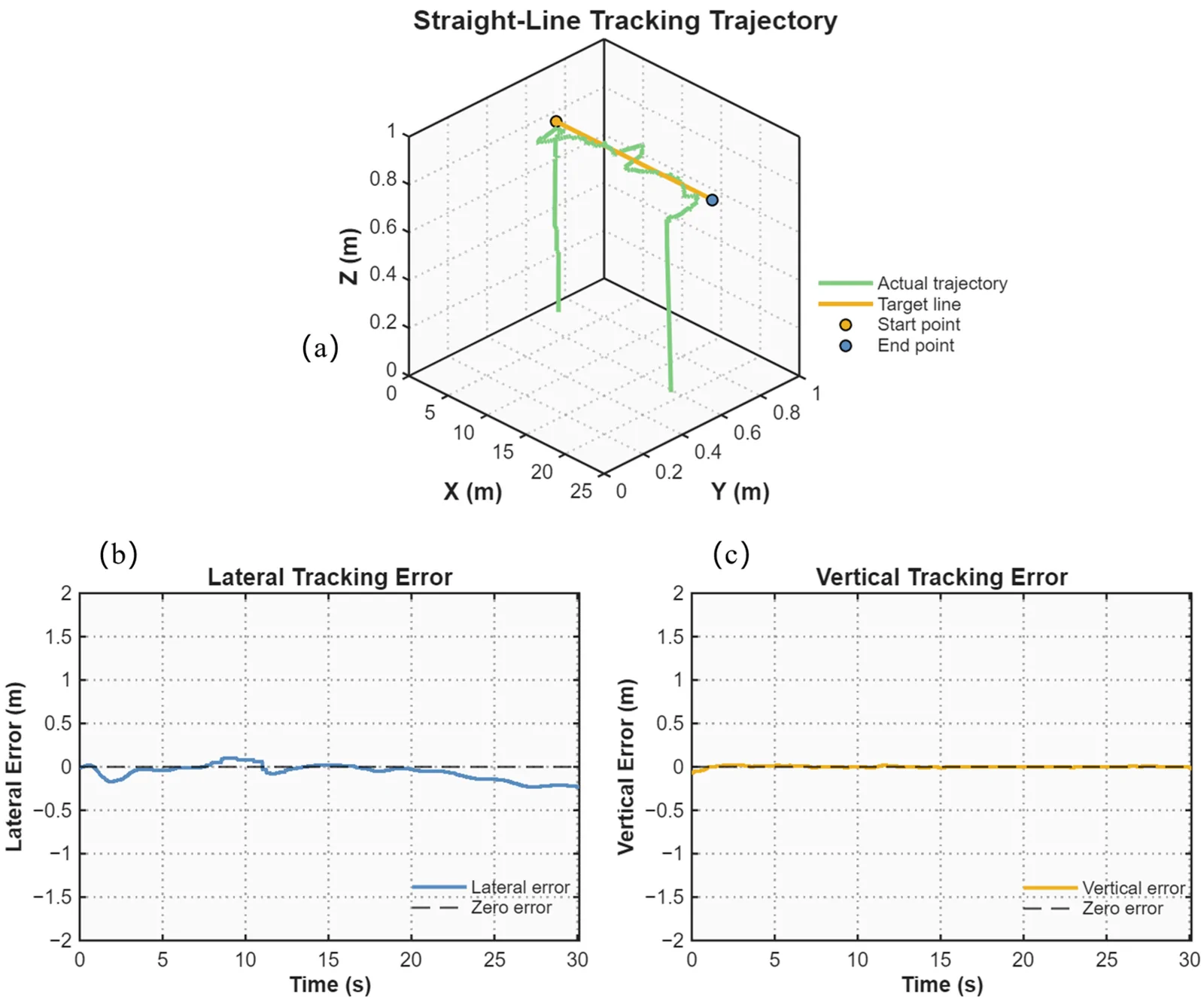
Straight-line tracking curve and lateral and vertical tracking errors of Test-3 trial: (**a**) 3D straight-line tracking trajectory and target straight line; (**b**) Lateral tracking error; (**c**) Vertical tracking error.

Figure 8a compares the three-dimensional target trajectory and the actual flight trajectory in one straight-line tracking experiment (Test-3 trial). As shown in Figure 8a and Supplementary Video S1, the aircraft was able to complete autonomous flight along the predefined straight-line direction. The actual trajectory was generally distributed near the target line, and no obvious divergence occurred. The lateral error curve in Figure 8b shows that the aircraft had a certain degree of left-right oscillation during straight-line tracking. However, the error remained within a bound range and did not continuously increase with flight time. These oscillations may be attributed to the closed-loop response characteristics of the cascaded PID controller and the dynamic response of the lightweight FWMAV. During trajectory tracking, the controller continuously adjusts the aircraft position according to the tracking error, and the finite response of the actuators may lead to repeated corrections around the desired trajectory. In addition, outdoor disturbances, such as wind fluctuations, and measurement noise from low-cost MEMS/GNSS sensors may contribute to short-term error variations. For this Test-3 trial shown in Figure 8, the lateral and vertical tracking error RMSE values were 0.1051 m and 0.0111 m, respectively. The vertical error curve in Figure 8c was generally close to zero, indicating that the aircraft could maintain the desired altitude during straight-line flight. The corresponding maximum lateral and vertical tracking errors were 0.23 m and 0.07 m, respectively.

Table 3 shows straight-line trajectory tracking results for three independent trials. Across the three trials, the average lateral and vertical RMSE values were 0.1112 m and 0.0222 m, respectively. The average maximum lateral and vertical tracking errors were 0.28 m and 0.10 m, respectively. The consistent tracking performance across the repeated trials indicates that the proposed system can achieve repeatable short-range outdoor trajectory tracking under the tested conditions.

Considering the effects of periodic aerodynamic fluctuations, outdoor wind disturbances, and short-term positioning noise from the low-cost GNSS module, the actual trajectory is difficult to fully coincide with the target straight line. Nevertheless, these results indicate that the proposed system can support outdoor straight-line autonomous tracking of the FWMAV under low-cost MEMS/GNSS fusion localization.

#### 3.2.3. V-Shaped Trajectory Flight Experiment

The straight-line tracking experiments were conducted at an approximately constant altitude. They mainly evaluated the horizontal trajectory tracking capability of the system. Therefore, a V-shaped altitude-varying trajectory flight experiment was conducted to evaluate the tracking capability during simultaneous horizontal and vertical motion (with the X and Z positions changed).

In this experiment, a three-dimensional reference trajectory containing descending and ascending segments was predefined. The trajectory consisted of an initial level forward-flight segment followed by a V-shaped altitude-varying flight segment. During the V-shaped flight segment, the aircraft continued to move forward while descending to the lowest point of the trajectory, and then continued forward while climbing back toward the original altitude.

**Figure 9:**
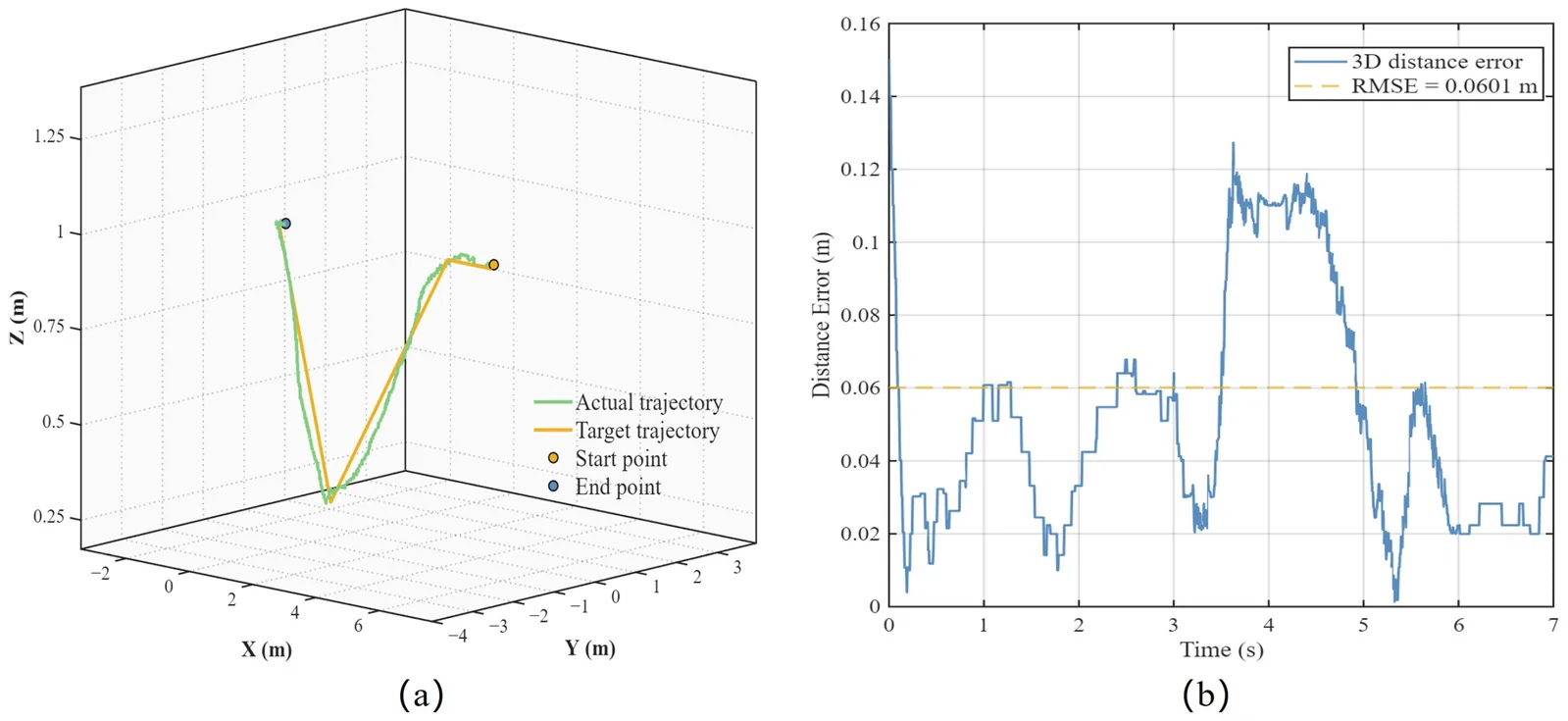
V-shaped trajectory tracking results: (**a**) Comparison between the estimated flight trajectory and the predefined target trajectory; (**b**) Three-dimensional spatial tracking error relative to the target trajectory.

Figure 9a compares the predefined V-shaped reference trajectory with the estimated flight trajectory. As shown in Figure 9a and Supplementary Video S1, the aircraft followed the overall V-shaped profile. It completed both the descending and ascending segments. The actual trajectory showed some deviation from the reference trajectory. However, no obvious divergence occurred. The RMSE of the three-dimensional distance between the estimated trajectory and the reference trajectory was 0.0601 m (Figure 9b). This value reflects the tracking deviation between the estimated state and the commanded trajectory. It does not represent the absolute flight-position error. The remaining deviation may be related to outdoor wind disturbances. It may also be related to the tuning and transient response of the vertical PID loop.

This experiment demonstrates coupled horizontal and vertical trajectory tracking. The X and Z positions changed simultaneously during the V-shaped motion. The Y position remained approximately constant. Therefore, this experiment provides evidence of altitude-varying spatial trajectory tracking.

## 4. Discussion

The above experimental results show that the proposed low-cost MEMS/GNSS fusion navigation and cascaded PID control method can support outdoor position holding and straight-line trajectory tracking of the tailless FWMAV. Existing autonomous tailless FWMAVs often rely on motion capture systems, UWB infrastructure, or vision-based navigation methods. In contrast, the proposed approach reduces the requirements for payload capacity and onboard computing resources. This work provides a practical step toward lightweight autonomous outdoor flight of FWMAVs under strict hardware constraints.

The ultimate goal of this research is to achieve fully autonomous outdoor flight of tailless FWMAVs in complex real-world environments. The current system demonstrates the feasibility of lightweight onboard navigation and control under open outdoor conditions. However, several aspects still require further improvement before reliable long-duration autonomous operation can be achieved in more challenging scenarios.

First, the autonomous flight experiments lacked an independent positioning reference. This limits the interpretation of the reported trajectory errors. Motion capture systems are difficult to deploy in outdoor environments. RTK-GNSS also introduces additional payload and hardware requirements, which are challenging for FWMAVs with a wingspan of less than 0.5 m. Therefore, the reported tracking errors represent the deviation between the estimated states and commanded trajectories. These results demonstrate the trajectory-tracking capability of the system rather than its absolute flight-position accuracy. Future work will incorporate independent positioning references. This will allow a more rigorous evaluation of absolute positioning performance.

Second, the current navigation framework still relies on GNSS availability. Its performance under GNSS-degraded conditions therefore requires further improvement. Buildings and vegetation may degrade GNSS signals in urban areas or forests. During short-term GNSS degradation or temporary signal loss, the ESKF can continue state propagation using IMU measurements. The barometer can also provide altitude constraints. However, position uncertainty gradually increases without GNSS position updates because of inertial integration drift.

In addition, the current system does not provide an absolute heading reference. Magnetometer measurements were disabled because of electromagnetic interference from the motor-driven flapping mechanism. Therefore, yaw estimation mainly relies on the integration of gyroscope signals. During translational flight, GNSS velocity measurements provide an indirect heading constraint. This constraint helps reduce yaw drift. However, it becomes weak during hovering or low-speed flight. The limited translational motion reduces heading observability. As a result, gyro bias and noise may cause gradual yaw drift. This drift may affect long-term position-holding performance. Notably, the straight-line tracking and V-shaped trajectory experiments conducted in this study were designed to maintain a constant yaw angle without intentional yaw rotation. These experimental results only validate that yaw divergence does not occur under the specific test conditions of this work. The influence of yaw dynamics on fully autonomous flight missions is beyond the scope of the current research and will be systematically investigated in future studies. Future work will investigate lightweight complementary sensors, such as optical flow and vision-based measurements. These sensors can provide additional position and heading constraints. They may also improve navigation robustness under GNSS-limited conditions.

Third, the vibration characteristics of the FWMAV have not yet been quantitatively analyzed. The current preprocessing and ESKF framework can reduce the influence of flapping-induced disturbances. However, periodic wing motion introduces high-frequency and non-stationary disturbances into the IMU measurements. These disturbances may affect attitude and state estimation performance. Future work will investigate vibration-aware filtering strategies. Frequency-domain analysis and adaptive filtering methods will be considered to further improve the robustness of state estimation.

Fourth, the disturbance rejection capability of the current controller requires further investigation under more complex outdoor conditions. The flight experiments were conducted under relatively favorable weather conditions, with the wind speed below 1.5 m/s. Under these conditions, the cascaded PID controller maintained stable position holding and trajectory tracking. These results demonstrate its effectiveness under mild external disturbances.

However, stronger crosswinds or gusts may reduce the tracking performance of the current controller. The tailless FWMAV has a lightweight structure and limited actuator capability. Therefore, the available aerodynamic force and control torque are constrained. The current controller compensates for position errors by generating attitude commands. These commands change the direction of the aerodynamic forces. However, external disturbances may exceed the available control authority. In this case, the attitude adjustment may be insufficient to compensate for the disturbance. This can lead to larger trajectory deviations. Future work will focus on improving disturbance rejection capability. Disturbance estimation and compensation strategies will be investigated. Adaptive control and disturbance-observer-based methods will also be considered. These methods may improve autonomous flight robustness under uncertain outdoor conditions without sacrificing the lightweight design.

Finally, the current experimental validation includes position holding, straight-line trajectory tracking at approximately constant altitude, and a V-shaped trajectory tracking involving simultaneous horizontal and vertical motion. The V-shaped experiment extends the validation beyond constant-altitude horizontal flight and provides evidence that the system can track altitude-varying spatial trajectories. However, the lateral position remained approximately constant during this experiment, and the trajectory did not include simultaneous changes in all three position axes or substantial directional changes. Therefore, the current results should not be interpreted as a complete evaluation of general three-dimensional autonomous navigation. Future experiments will investigate longer-duration and more complex waypoint-based trajectories involving changes in horizontal direction and altitude, together with repeated trials, to provide a more comprehensive evaluation of the three-dimensional autonomous navigation capability of the system.

## 5. Conclusions

This study developed and experimentally validated a lightweight onboard autonomous navigation and control system for a 33 cm-wingspan, 31 g tailless flapping-wing micro aerial vehicle. The proposed system integrates low-cost MEMS sensors (MPU9250 IMU and SP06 barometer), a GNSS module (BZ121), and a 1.9 g onboard flight-control board. It enables autonomous outdoor flight without relying on external motion-capture infrastructure or computationally intensive vision-based sensing. An error-state Kalman filter (ESKF) was employed to fuse inertial, GNSS, and barometric measurements. A cascaded PID controller was then designed to generate closed-loop control commands based on the estimated states without requiring an accurate aerodynamic model.

The proposed system was evaluated through outdoor experiments, including a ground-based validation experiment, autonomous position-holding flight, straight-line flight trajectory tracking, and V-shaped altitude-varying trajectory tracking. In the ground-based validation experiment, the estimated trajectory remained consistent with the overall GNSS route. In the position-holding experiment, the vehicle reached the commanded locations and remained around the active setpoint during the hovering segment without persistent drift. In the three independent straight-line flight trials, the vehicle progressed along the prescribed 20 m straight-line path, with the average maximum lateral and vertical tracking errors of 0.28 m and 0.10 m, respectively. In the V-shaped trajectory flight experiment, the vehicle completed both descending and ascending segments while continuing forward, and the three-dimensional distance RMSE between the ESKF-estimated trajectory and the predefined reference trajectory was 0.0601 m.

These results demonstrate the preliminary feasibility of combining low-cost MEMS/GNSS fusion and cascaded state-feedback control for outdoor autonomous flight of a tailless FWMAV under open-sky GNSS conditions. More importantly, the experiments show that the proposed onboard estimator can provide sufficiently continuous and stable state feedback to support closed-loop flight control despite the vibration, coupling, and disturbance characteristics of the tailless flapping-wing platform.

Nevertheless, the current experiments mainly validate the feasibility of outdoor position holding and short-range trajectory tracking under open-sky GNSS conditions. The absolute localization accuracy and robustness of the system under more complex outdoor environments require further investigation. Future work will introduce independent references, such as RTK GNSS or motion capture, to further evaluate estimation performance. Vibration-aware fusion, wind and disturbance estimation, and adaptive disturbance-rejection control will also be investigated. Lightweight complementary sensing methods, such as optical flow or vision, will be explored to maintain navigation performance in GNSS-degraded environments.

## Figures and Tables

**Table 1:** Reference values of sensor noise parameters.

Sensor	Parameter	Value	Source
Accelerometer	Noise density	$2.94\times 10^{-3}m/s^{2}/\sqrt{\mathrm{Hz}}$	[30]
Accelerometer	Bias instability	$1.06\times 10^{-3}m/s^{2}$	[32]
Gyroscope	Noise density	0.01°$/s/\sqrt{\mathrm{Hz}}$	[30]
Gyroscope	Bias instability	0.274°/s	[32]
Barometer	Pressure noise	0.3 Pa RMS	[31]

**Table 2:** PID gains of the cascaded position, velocity, angle and angular velocity controllers.

Channel	Loop	$K_{p}$	$K_{i}$	$K_{d}$	Loop	$K_{p}$	$K_{i}$	$K_{d}$
*x*	Velocity	75	0	0	Position	40	0	6
*y*	Velocity	75	0	0	Position	40	0	6
*z*	Velocity	800	0	40	Position	300	0	30
pitch	Angular velocity	600	0	100	Angle	75	10	40
roll	Angular velocity	400	0	30	Angle	110	0	10
yaw	Angular velocity	150	80	0	Angle	100	0	0

**Table 3:** Straight-line trajectory tracking results for three independent trials.

Trial	Lateral RMSE	Maximum Lateral Error	Vertical RMSE	Maximum Vertical Error
Test-1	0.0982 m	0.31 m	0.0231 m	0.13 m
Test-2	0.1303 m	0.29 m	0.0323 m	0.11 m
Test-3	0.1051 m	0.23 m	0.0111 m	0.07 m
Average	0.1112 m	0.28 m	0.0222 m	0.10 m

## Data Availability

Data will be made available on request.

## Supplementary Materials

The following supporting information can be downloaded at https://www.mdpi.com/article/10.3390/biomimetics11100725/s1, Video S1: Outdoor Tests Video.mp4.
